# Report of Deaths in Buffalo for the Months of October and November, 1851

**Published:** 1852-01

**Authors:** W. L. G. Smith

**Affiliations:** Clerk


*Report of Deaths in Buffalo for the Months of October and November*, 1851.

BOARD OF HEALTH, BUFFALO, Nov. 15th, 1851.

Number of deaths reported to the Board of Health in the month of October last:—Males, 49; Females, 20—total, 69. Adults, 30; Infants, 39.

*Diseases.* Diarrhœa, 7; pneumonia, 7; convulsions, 3; consumption, 13; unknown, 5; hydrocephalus, 3; stillborn, 1; cholera morbus, 1; scrofula, 1; whooping cough, 2; fracture of the skull, 1; scarlet fever, 2; typhus fever, 5; disease of the heart, 1; inflammation of the bladder, 1; cancer, 1; dysentery, 6; inflammation of the bowels, 1; imperfect organization, 1; bronchitis, 1; dropsy of the chest, 1; casualty, 1; debility, 1; anemia, 2.

---

Dec. 11th, 1851.

Number of deaths reported to the Board of Health in the month of November last: Males, 44; Females, 40; total, 84. Adults, 45; children, 39.

*Diseases.* Diarrhœa, 3; puerperal fever, 2; convulsions, 3; consumption, 12; unknown, 7; hydrocephalus, 3; stillborn, 3; cholera infantum, 1; small pox, 1; whooping cough, 2; summer complaint, 1; scarlet fever, 5; croup, 5; pleurisy, 1; typhus fever, 9; disease of the heart, 2; inflammation of the brain, 1; cancer, 2; dysentery, 3; inflammation in the bowels, 3; inflammation of the lungs, 4; bronchitis, 2; dropsy, 2; casualty, 3; debility, 1; old age, 2; albuminaria, 2; poison, 1; drowned, 1.

By order of the Board.

W. L. G. SMITH, *Clerk.*

